# Toxicological evaluation of porcine bile powder in Kunming mice and Sprague–Dawley rats

**DOI:** 10.3389/fphar.2024.1424940

**Published:** 2024-07-08

**Authors:** Lirong Wu, Jieyi Wang, Jing Lei, Kun Ge, Chun Qu, Jiajian Liu, Fengjie Huang, Dongnan Sun, Xiaowen Chao, Tianlu Chen, Aihua Zhao, Wei Jia, Xiaojiao Zheng, Guoxiang Xie

**Affiliations:** ^1^ Center for Translational Medicine and Shanghai Key Laboratory of Diabetes Mellitus, Shanghai Sixth People’s Hospital Affiliated to Shanghai Jiao Tong University School of Medicine, Shanghai, China; ^2^ Human Metabolomics Institute, Inc., Shenzhen, China; ^3^ Department of Pharmacology and Pharmacy, University of Hong Kong, Hong Kong, Hong Kong SAR, China

**Keywords:** porcine bile powder, chemical composition, acute oral toxicity, subchronic oral toxicity, genotoxity, teratogenicity

## Abstract

**Background:** Porcine bile powder (PBP) is a traditional Chinese medicine that has been used for centuries in various therapeutic applications. However, PBP has not previously undergone comprehensive component analysis and not been evaluated for safety through standard *in vivo* toxicological studies.

**Methods:** In our study, we characterized the component of PBP by liquid chromatography-mass spectrometry. The acute and subchronic oral toxicity, genotoxicity, and teratogenicity studies of PBP were designed and conducted in Kunming mice and Sprague-Dawley (SD) rats.

**Results:** The chemical analysis of PBP showed that the main components of PBP were bile acids (BAs), especially glycochenodeoxycholic acid. There were no signs of toxicity observed in the acute oral test and the subchronic test. In the genotoxicity tests, no positive results were observed in the bacterial reverse mutation test. Additionally, in the mammalian micronucleus test and mouse spermatocyte chromosomal aberration test, no abnormal chromosomes were observed. In the teratogenicity test, no abnormal fetal development was observed.

**Conclusion:** Our findings demonstrate that PBP, composed mainly of BAs, is non-toxic and safe based on the conditions tested in this study.

## 1 Introduction

Porcine bile powder (PBP), also termed *PULVIS FELLIS SUIS*, is a traditional Chinese medicine recorded in Chinese Pharmacopoeia. It is a dried product derived from pig bile, characterized by a yellowish-brown appearance, bitter taste, and strong odor. The application of PBP has a long history, with its medicinal value traced back to ancient China. As early as the Eastern Han Dynasty, the *Treatise on febrile diseases* written by Zhang Zhongjing contained some Chinese medical prescriptions using pig bile. Currently, PBP has been commonly used for treating gastrointestinal, respiratory, and skin disorders, such as cough, asthma, constipation, dysentery, and boils (Qiao et al., 2011; China Medical Science and Technology Publishing Co, 2020). A recent study showed that PBP could relieve airway inflammation by decreasing the levels of interleukin (IL)-4, IL-5, and IL-13 produced by T-helper (Th) 2 cells, and increasing the level of interferon (IFN)-γ produced by Th1 in bronchoalveolar lavage fluid (BALF) (He et al., 2017). PBP can also relieve intestinal inflammation by inhibiting the expression of cyclooxygenase-2 protein and the accumulation of myeloperoxidase in the intestine (He et al., 2011).

In addition to the traditional efficacy, PBP attracted attention in its role in treating other diseases such as diabetes, non-alcoholic steatohepatitis, depression, dementia, and stroke (Zheng et al., 2021a; Fuchs and Trauner, 2022; Lirong et al., 2022; Kuang et al., 2023) with the progress in the research of bile acids (BAs). BAs are increasingly being considered metabolic integrators and signaling molecules, which can regulate metabolic processes, such as triglyceride, cholesterol, and glucose homeostasis (Thomas et al., 2008; Jia et al., 2020; Li et al., 2022). For example, hyocholic acid (HCA) and its chemical derivatives including hyodeoxycholic acid (HDCA), glycohyocholic acid (GHCA), taurochenodeoxycholic acid (THCA), glycohyodeoxycholic acid (GHDCA), and taurohyodeoxycholic acid (THDCA) are the major BAs in PBP (Zheng et al., 2021b). Previous studies showed that HCA species not only improved glucose homeostasis by affecting glucagon-like peptide 1 (GLP-1) secretion through interaction with the intestinal receptors Takeda G protein-coupled receptor 5 and farnesoid X receptor in diabetic mice but were also strongly negatively correlated with diabetes and glycemic markers in several clinical cohorts (Zheng et al., 2021a).

PBP has been officially recorded in the Chinese Pharmacopoeia, however, as an ancient experiential effective prescription, there is a lack of systematic studies on its components and safety. In this study, we characterized the main components of PBP and further assessed its potential toxicity through acute, subchronic toxicity, genotoxicity, and teratogenicity testing, in order to identify safe doses of PBP according to the National Food Safety Standard (GB 15193).

## 2 Materials and methods

### 2.1 Materials

PBP (Lot #G20210901) was provided by Henan Liwei Biological Pharmaceutical Co., Ltd. (Jiaozuo, Henan, China).

Chemical composition of PBP was analyzed via liquid chromatography–triple quadrupole mass spectrometry (LC-TQMS) using the Q300 Metabolite Assay Kit (Human Metabolomics Institute, Inc., Shenzhen, Guangdong, China). Hematological indicators were measured using blood cell analysis reagents and automatic blood analyzer BC-5000Vet (Shenzhen Mindray Medical International Co., Ltd., Shenzhen, Guangdong, China). Blood biochemical indices were determined using biochemical analysis reagents and the automatic biochemical analyzer BS-330E (Shenzhen Mindray Medical International Co., Ltd.). The coagulation index was determined using hemagglutination reagents and the automatic hemagglutination meter STA Compact Max (Stago Diagnosis Technology (Tianjin) Co., Ltd., Tianjin, China). The urine index was measured using urinalysis reagents and the urine analyzer URIT-500B (URIT MEDICAL Electronic Co., Ltd., Guilin, Guangxi, China). Cyclophosphamide was purchased from Ron Biotechnology Co., Ltd. (Guangzhou, Guangdong, China). 2-Aminofluorene, 1,8-dihydroxyanthraquinone, 2-aminoanthracene, sodium azide, dexon, and 9-aminoacridine were provided by Solarbio Science & Technology Co., Ltd. (Beijing, China), Kuer Biotechnology Co., Ltd. (Hefei, Anhui, China), Shanghai Macklin Biochemical Co., Ltd. (Shanghai, China), Fuchen (Tianjin) Chemical Reagent Co., Ltd. (Tianjin, China), Accustandard, Inc. (New Haven, CT, United States), and Aladdin Reagent (Shanghai) Co., Ltd. (Shanghai, China), respectively.

### 2.2 Animals and bacteria

Specific-pathogen-free (SPF) Kunming mice (5–9 weeks old, 28.9–33.9 g) and SPF-grade Sprague–Dawley (SD) rats (5–9 weeks old, 59–86 g) were provided by SPF Biotech Co., Ltd. (Beijing, China; animal production license No. SCXK [Jing] 2019-0010). *Salmonella typhimurium* histidine-auxotrophic strains TA98, TA100, and TA102 were provided by Qiyi Biotech Co., Ltd. (Shanghai, China), and TA1535 and TA1537 were provided by Bioplus Biotech Co., Ltd. (Shanghai, China).

All mice and rats were acclimated for 3–5 days and housed in an SPF environment under controlled conditions of a 12 h light/12 h dark cycle at 20°C–22°C and 45% ± 5% humidity, with free access to purified rodent diet and ultrapure water. All animal tests were designed in accordance with the requirements of the National Food Safety Standard (GB 15193) for toxicity tests. All animal studies were approved by the Institutional Animal Care and Use Committee at Xi’an United Nations Quality Detection Group Co., Ltd. (UNQD, Xi’an, China). The ethics approval number for the use of animals in this study is AUP-TOX-20210802004.

### 2.3 Chemical composition analysis

Chemical composition analysis of PBP was conducted. PBP was diluted in water and prepared as a 250 mg/mL solution. A 20-μL aliquot of the PBP solution was added to each well of a 96-well plate. Then, the plate was transferred to a Biomek 4000 workstation. Each well was supplemented with ice-cold methanol with partial internal standards. After intense vortexing for 5 min, the plate was centrifuged at 4,000 ×g for 30 min. Then, the plate was put into the workstation and 30 μL of supernatant was transferred to another 96-well plate with 20 μL of freshly prepared derivative reagent. The plate was sealed and derivatized at 30°C for 60 min. When the derivatization was complete, 350 μL of ice-cold 50% methanol solution was added per well, followed by storage at −20°C for 20 min. After storage at −20°C, the plate was centrifuged at 4,000 ×g for 30 min. A total of 135 μL of supernatant was transferred to a new 96-well plate with 15 μL of internal standards per well. Serial dilutions of derivatized stock standards were added to the remaining wells. The plate was then sealed and subject for LC-TQMS (ACQUITY UPLC-Xevo TQ-S, Waters, Milford, MA, United States) analysis according to our previously established protocol (Xie et al., 2021). All chromatographic separations were performed with an ACQUITY BEHC18 column (1.7 mm, 100 mm × 2.1 mm internal dimensions; Waters, Milford, MA, United States). The mobile phase consisted of 0.1% formic acid in LC–MS grade water (mobile phase A) and 0.1% formic acid in LC–MS grade acetonitrile (mobile phase B) and run at a flow rate of 0.4 mL/min. The gradient was programmed as follows: 0–1 min (5% B), 1–5 min (5%–25% B), 5–15.5 min (25%–40% B), 15.5–17.5 min (40%–95% B), 17.5–19 min (95%B), 19–19.5 min (95%–5% B), and 19.6–21 min (5% B). The column was maintained at 45°C. The mass spectrometer was operated in negative ion mode with a capillary voltage of 1.2 kV. The source temperature is 150°C, and the desolvation gas temperature is 550°C. The data were collected with multiple reaction monitor (MRM) mode.

### 2.4 Acute oral toxicity test

For the acute oral toxicity test, 10 male and 10 female Kunming mice were used (certification no. 110324210105842634). The 20 mice were administered a single dose of PBP by gavage at 15 g/kg body weight (BW) after 16 h of fasting. After gavage, the animals were continuously observed for toxicity symptoms and mortality for 14 days. On day 14, the animals were anesthetized by inhalational isoflurane for a gross autopsy.

### 2.5 Subchronic toxicity test

Forty male and forty female SPF-grade SD rats (certification no. 110324220102434852) were used for a 90-day oral toxicity test. Eighty rats were randomly divided into four groups containing equal numbers of males and females, including three dose groups (PBP at doses of 2.25 g/kg BW, 1.50 g/kg BW, and 0.75 g/kg BW) and a solvent control group (pure water). The mice were administered with PBP or solvent for 6 days a week by gavage. The animals were provided free access to food and water during the test. The general performance, behavior, poisoning symptoms, and mortality of the animals were observed every day. BW and food consumption were recorded weekly. At the end of the test, the rats were fasted for 16 h, after which the BW was determined, and blood was collected from the abdominal aorta after anesthesia. The hematological, blood biochemical, coagulation, and urine indices were measured. Finally, the rats were executed in the 13th week and pathological changes of the organs were observed, including liver, kidney, spleen, adrenal gland, brain, heart, thymus, uterus, ovaries, testes, and epididymis. Their weights were also recorded to calculate the organ weight/BW ratio as the relative organ weight.

### 2.6 Genotoxicity experiments

#### 2.6.1 Bacterial reverse mutation test

Five *S. typhimurium* histidine-auxotrophic strains, TA98, TA100, TA102, TA1535, and TA1537, were used for testing toxicity under conditions with or without the *in vitro* metabolic activation system from rat livers (S9 mixture, 0.5 mL/plate). The plate incorporation method was employed. The test included dose groups (PBP at doses of 5,000, 1,581, 500, 158, and 50 μg/plate), solvent control groups (sterilized pure water), spontaneous reflux groups, and positive control groups. In the test with S9, 2-aminofluorene (20.0 μg/plate) was used as a positive control of TA98 and TA100, 1,8-dihydroxyanthraquinone (50.0 μg/plate) was used as a positive control of TA102, and 2-aminoanthracene (2.0 μg/plate) was used as a positive control of TA1535 and TA1537. In the test without S9, sodium azide (1.5 μg/plate) was used as a positive control of TA100 and TA1535, dioctosone (50.0 μg/plate) was used as a positive control of TA98 and TA102, and 9-aminoacridine (50.0 μg/plate) was used as a positive control of TA1537. Each test dose was applied to three parallel plates. The number of revertant colonies was recorded upon incubation at 37°C for 48 h.

#### 2.6.2 Micronucleus test

A total of 25 male and 25 female Kunming mice (certification no. 110324210106489656) were used for the micronucleus test. The 50 Kunming mice were randomly divided into five groups of equal numbers of males and females (*n* = 5 per sex per group), including dose groups (PBP at doses of 10.0 g/kg BW, 5.0 g/kg BW, and 2.5 g/kg BW), a solvent control group (pure water), and a positive control group (cyclophosphamide at a dose of 40 mg/kg BW). The mice were administered with PBP, solvent, or positive control by gavage twice at an interval of 24 h. The mice were euthanized at the 6th h after the second gavage, and femoral bone marrow smears were taken for fixation, Giemsa staining, and inspection by oil microscopy. The number of micronucleated cells among 2,000 polychromatic erythrocytes (PCEs) per mouse was analyzed to determine the micronucleation ratio. Additionally, 200 red blood cells (RBCs) were observed and the ratio of PCEs to total RBCs was determined.

#### 2.6.3 Spermatocyte chromosomal aberration test

Thirty male Kunming mice (certification no. 110324210106489656) were used for the mouse spermatocyte chromosomal aberration test. These mice were randomly divided into five groups (high-dose group with 10 mice; the other groups with 5 mice), including dose groups (PBP at doses of 10.0 g/kg BW, 5.0 g/kg BW, and 2.5 g/kg BW), a solvent control group (pure water), and a positive control group (intraperitoneal [i.p.] injections with CP at a dose of 40 mg/kg BW). The mice administered with PBP or solvent once by gavage. Animals in the high-dose group were euthanized at two timepoints, 24 and 48 h after the administration of PBP, with five animals at each point. The remaining groups of animals were euthanized 24 h after the administration of PBP. All animals were injected with colchicine (i.p., with 6 mg/kg BW) 5 h before being euthanized. The testicles were extracted, the membrane was removed, and the seminiferous tubules were separated. After the procedure of fixation, centrifugation, and Giemsa staining, the samples were observed under a microscope. The type, number of chromosomal aberrations, and rate of aberrant cells in 100 metaphase dividing phase cells per mouse were recorded, and 1,000 cells per animal were observed to determine the spermatogonia mitotic index under an oil microscope.

### 2.7 Teratogenicity testing

A total of 88 female and 80 male SPF-grade SD rats (certification no. 110324220105057957 [female]; no. 110324220105058082 [male]) were used for teratogenicity testing. The male and female rats were caged 1:1, and the next morning the presence of a pubic plug was checked to determine the fertilized rats. The fertilized rats were weighed, numbered, and the date was recorded as day 0 of conception. The fertilized rats were then randomly divided into dose groups (PBP at doses of 2.25 g/kg BW/day, 1.50 g/kg BW/day, and 0.75 g/kg BW/day) and a solvent control group (pure water). PBP was administered on days 6–15 after conception by gavage once a day. Maternal weight was determined on days 0, 6, 9, 12, 15, and 20 after conception. The poisoning status of the fertilized rats was observed and recorded daily. The dams were sacrificed on the day before delivery, the pregnancy and fetal status were examined by laparotomy, and the uterus was removed to weigh the uterine conjoined fetus. The numbers of corpora lutea, premature stillbirths, late stillbirths, live births, and implantations were recorded. The fetal rats were taken out and their sex, weight, body length, and fetal appearance were recorded, along with checking for abnormalities. Half of the fetal rats in each litter were examined for internal organs and bones.

### 2.8 Experimental statistics

Data were analyzed using SPSS 24 statistical software. All results are presented as mean ± SD. Qualitative data were compared between groups using χ^2^ test, while quantitative data were compared using Student’s t-test. A significance level of *p* < 0.05 was considered statistically significant.

## 3 Results

### 3.1 Composition analysis

**FIGURE 1:**
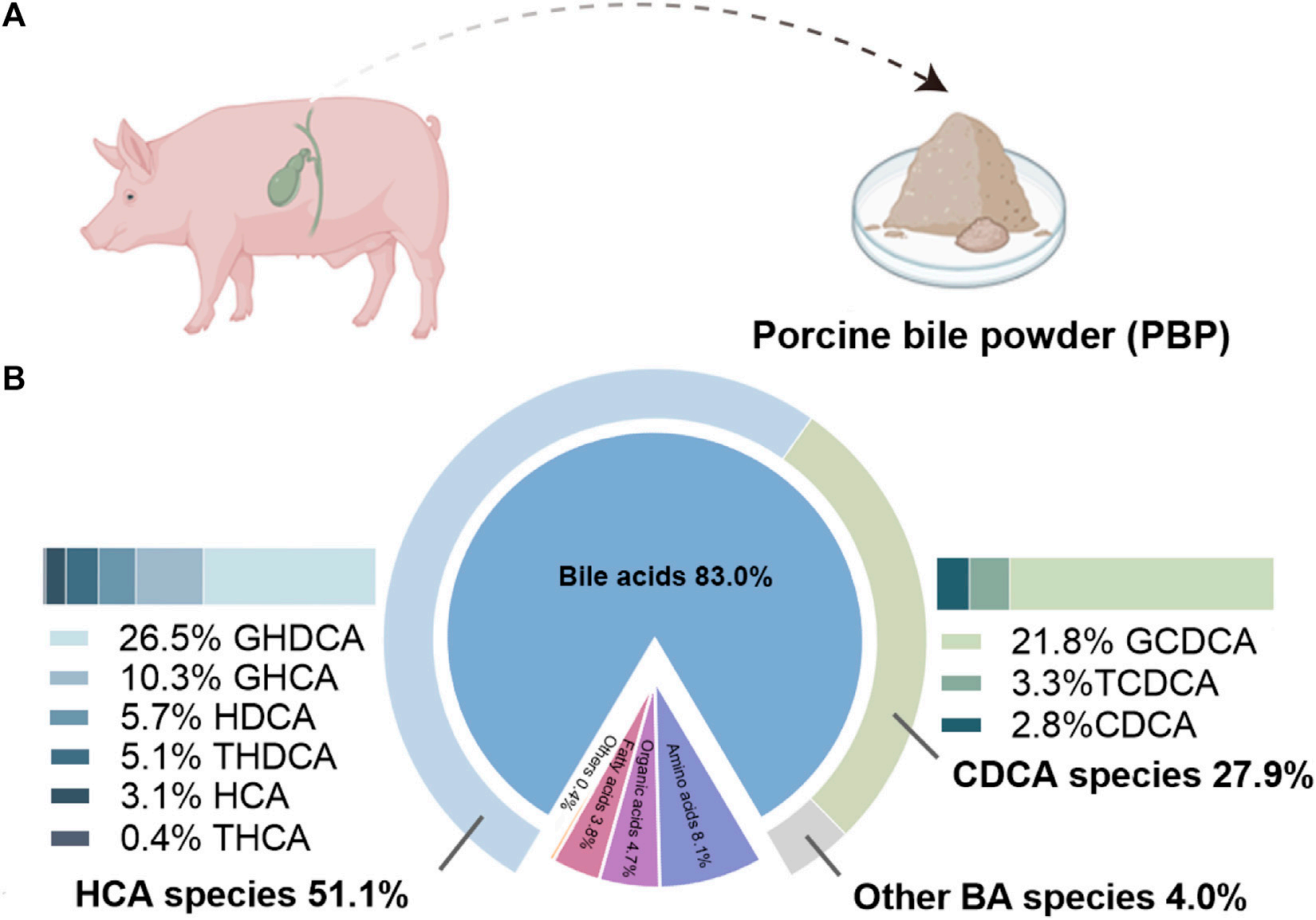
Source **(A)** and components **(B)** of PBP.

The typical total ion chromatograms of all metabolites identified in PBP and extracted ion chromatograms of BAs were shown in Supplementary Figures S1A, B. PBP mainly included BAs (83.0%), amino acids (8.1%), organic acids (4.7%), fatty acids (3.8%), and other components (0.4%). BAs were found to be the main components of PBP. The BAs detected in the PBP can be simply divided into three species. The most abundant was the HCA species, accounting for 51.1%, which included 26.5% GHDCA, 10.3% GHCA, 5.7% HDCA, 5.1% THDCA, 3.1% HCA, and 0.4% THCA. The second most abundant was the chenodeoxycholic acid (CDCA) species, accounting for 27.9%, including 21.8% glycochenodeoxycholic acid (GCDCA), 3.3% taurochenodeoxycholic acid (TCDCA), and 2.8% CDCA. The remaining BA species accounted for 4.0% (Figure 1).

### 3.2 Safety analysis

#### 3.2.1 Acute oral toxicity test in mice

To determine the half lethal concentration of PBP, an acute oral toxicity test was performed in mice. In the 14-day acute toxicity test of mice, no obvious poisoning symptoms were found in the animals, and no animals died after 14 days of observation at a dose of 15 g/kg BW (Table 1). At the end of the test, the animals were dissected, and no clear abnormal changes were found in the liver, kidney, spleen, heart, lung, stomach, intestines, or other major organs. According to the acute toxic dose classification (the National Food Safety Standard [GB 15193]), PBP was found to be nontoxic at experimental doses.

**TABLE 1:** Results of acute oral toxicity test in mice.

Sex	Number of animals	Dose (g/kg.BW)	Initial weight (g)	Day-7 weight (g)	Day-14 weight (g)	Number of animals with symptoms of toxicity	Number of deaths	LD50 (g/kg.BW)
Female	10	15.0	20.9 ± 0.9	28.8 ± 2.1	34.1 ± 2.3	0	0	>15.0
Male	10	15.0	21.2 ± 0.6	30.9 ± 1.2	38.1 ± 1.4	0	0	>15.0

LD50 is a test substance dose that can cause animal mortality of 50% after once/multiple administrations of PBP, by oral gavage/within 24 h. Data are shown as mean ± SD.

#### 3.2.2 Subchronic oral toxicity test in rats

**FIGURE 2:**
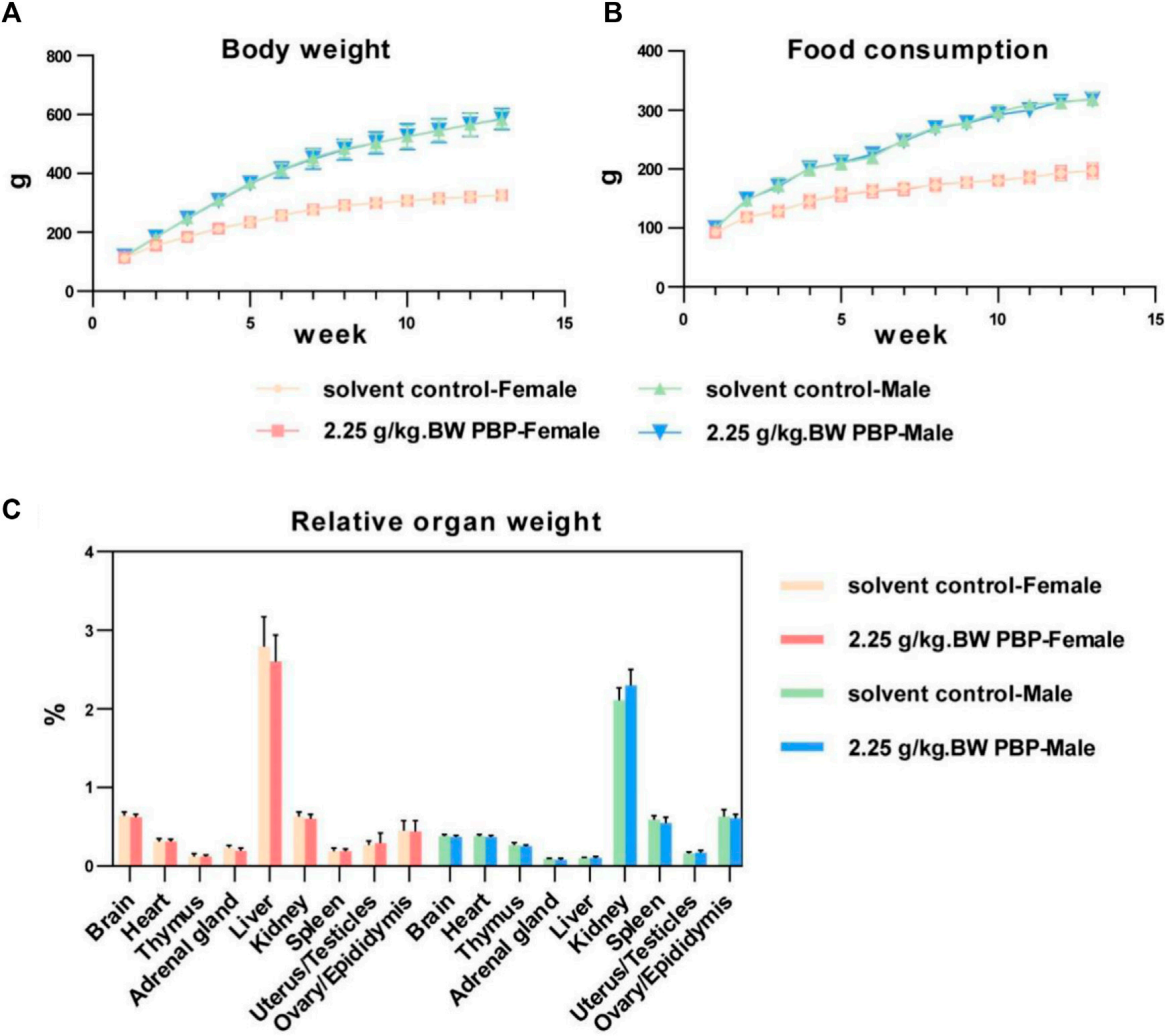
Subchronic oral toxicity test. **(A)** Body weights of rats during 1–13 weeks of intervention; **(B)** Food consumption of rats during 1–13 weeks of intervention; **(C)** Relative organ weight (organ weight/body weight).

To assess the toxicity of PBP on the important organs and physiological functions of the body, a subchronic toxicity test was performed and the test dose was estimated from the acute toxicity test. Throughout the study period, the BW of the rats continued to increase steadily, and no signs of toxicity or mortality were observed. No significant differences were observed between the solvent control group and the various dose groups in terms of initial BW, weekly BW, total BW gain, fasting BW, weekly food consumption, and food utilization rate for both male and female rats (*p* > 0.05, Figure 2; Supplementary Tables S1–S3). Similarly, there were no significant differences in organ weight and relative organ weight within each dose group (*p* > 0.05, Figure 2; Supplementary Table S4). Hematological test results, blood biochemical detection results, and urine test results for male and female rats in each dose group also did not show any significant differences (*p* > 0.05, Supplementary Tables S5–S7).

#### 3.2.3 Genotoxicity test

##### 3.2.3.1 Bacterial reverse mutation test (Ames test)

Bacterial reverse mutation tests were conducted to evaluate the mutagenic potential of PBP. Compared with the spontaneous revertant group and solvent control, the number of *S. typhimurium* (TA98, TA100, TA102, TA1535, TA1537), with or without the addition of S9, did not exceed twice the number of spontaneous revertant colonies in each dose group, and there was also no dose–response relationship according to the National Food Safety Standard (GB 15193). These results indicated that the mutagenesis test result of PBP was negative. Thus, PBP does not cause genetic mutations at the tested concentrations (Table 2).

**TABLE 2:** Results of bacterial reverse mutation test.

Dose		Number of colonies per dish				
(μg/plate)	S9	TA98	TA100	TA102	TA1535	TA1537
50	−	34.0 ± 2.0	136.7 ± 7.1	265.0 ± 8.7	13.7 ± 1.5	9.3 ± 1.2
158	−	35.3 ± 2.1	134.3 ± 8.5	259.0 ± 9.2	12.3 ± 2.3	10.3 ± 1.5
500	−	33.7 ± 1.5	138.0 ± 7.2	258.0 ± 9.6	13.3 ± 1.5	9.0 ± 2.6
1,581	−	35.7 ± 2.1	136.3 ± 6.7	261.7 ± 8.4	14.0 ± 1.7	10.7 ± 1.5
5,000	−	34.3 ± 2.5	135.0 ± 8.0	262.3 ± 9.6	12.7 ± 2.1	11.0 ± 1.7
Spontaneous revertant	−	34.7 ± 1.5	137.0 ± 7.5	260.7 ± 8.7	13.0 ± 2.0	8.7 ± 1.5
Solvent control	−	35.3 ± 1.5	138.3 ± 6.8	259.3 ± 7.8	13.7 ± 1.5	9.3 ± 1.2
Positive control	−	1,838.7 ± 123.9	3,110.7 ± 180.9	966.3 ± 64.1	752.3 ± 44.0	178.3 ± 9.0
50	+	34.7 ± 1.5	137.3 ± 6.0	266.0 ± 9.2	13.7 ± 1.2	9.3 ± 1.5
158	+	34.0 ± 1.7	135.3 ± 8.6	260.3 ± 8.5	13.0 ± 2.0	11.0 ± 1.0
500	+	33.7 ± 2.1	138.3 ± 6.8	259.0 ± 8.5	14.0 ± 2.0	9.3 ± 1.2
1,581	+	36.0 ± 2.0	136.7 ± 6.4	262.0 ± 9.8	14.3 ± 1.5	11.0 ± 1.7
5,000	+	36.3 ± 1.5	137.7 ± 7.0	258.3 ± 9.3	13.0 ± 2.0	10.7 ± 1.5
Spontaneous revertant	+	35.0 ± 2.6	139.3 ± 7.0	261.3 ± 10.0	13.3 ± 1.5	8.7 ± 2.3
Solvent control	+	35.7 ± 1.5	138.7 ± 8.6	264.3 ± 8.5	14.0 ± 1.7	10.0 ± 1.0
Positive control	+	4,118.0 ± 232.4	2,792.0 ± 134.0	968.3 ± 50.5	195.3 ± 11.0	86.7 ± 9.1

Data are shown as the mean ± SD of three Petri dishes.

##### 3.2.3.2 Micronucleus test

A micronucleus test was conducted to assess whether PBP causes chromosomal damage and thus evaluate the mutagenicity of PBP. First, compared with the solvent control group, the micronucleus rate of the positive control group significantly increased (Supplementary Table S8, *p* < 0.01), while the micronucleus rate of bone marrow cells did not show a significant change in each dose group. Second, there was no significant difference in PCE/RBC values between each dose group and the solvent control group, and the proportion of PCEs among the total number of red blood cells in each dose group was not <20% of the solvent control group (Supplementary Table S8, *p* > 0.05). According to the National Food Safety Standard (GB 15193), these results showed that there was a negative result in the mammalian micronucleus test of PBP.

##### 3.2.3.3 Mouse spermatocyte chromosomal aberration test

A mouse spermatocyte chromosomal aberration test was conducted to assess whether PBP causes chromosomal damage to germ cells and evaluate its mutagenicity. Compared with that in the solvent control group, the rate of aberrant cells was significantly higher in the positive control group (Table 3, *p* < 0.01), while the rate was not significantly different between the dose groups and solvent control group (Table 3, *p* > 0.05). According to National Food Safety Standard (GB 15193), the mitotic index of spermatogonia in the high-dose group was not <50% of the index in the solvent control, indicating that PBP did not affect mitosis. The main types of chromosomal aberrations in the positive control group were breakage, fragmentation, and microsome, and there was no significant difference in the total number of cells with chromosomal aberration between the dose groups and the solvent control group (Supplementary Table S9). These results showed that the mouse spermatogonial chromosomal aberration test of PBP provided a negative result.

**TABLE 3:** Results of chromosomal aberration test of spermatogonia in mice.

Dose (g/kg.BW)	Number of animals examined	Number of cells examined	Mitotic index (%)	Number of metaphase cells examined	Number of aberrant cells	Rate of aberrant cells (%)
10.0 (24 h)	5	5,000	26.6 ± 1.7	500	2	0.4 ± 0.6
10.0 (48 h)	5	5,000	26.4 ± 1.8	500	1	0.2 ± 0.5
5.0	5	5,000	27.0 ± 1.9	500	1	0.2 ± 0.5
2.5	5	5,000	26.9 ± 1.7	500	1	0.2 ± 0.5
0.0	5	5,000	26.6 ± 1.9	500	2	0.4 ± 0.9
Positive control	5	5,000	26.7 ± 1.7	500	26	5.2 ± 0.8^**^

Data are shown as mean ± SD. ** indicates a significant difference (*p* < 0.01) compared with the solvent control group. A dose of “0.0” indicates the solvent control group.

#### 3.2.4 Teratogenicity experiment

**FIGURE 3:**
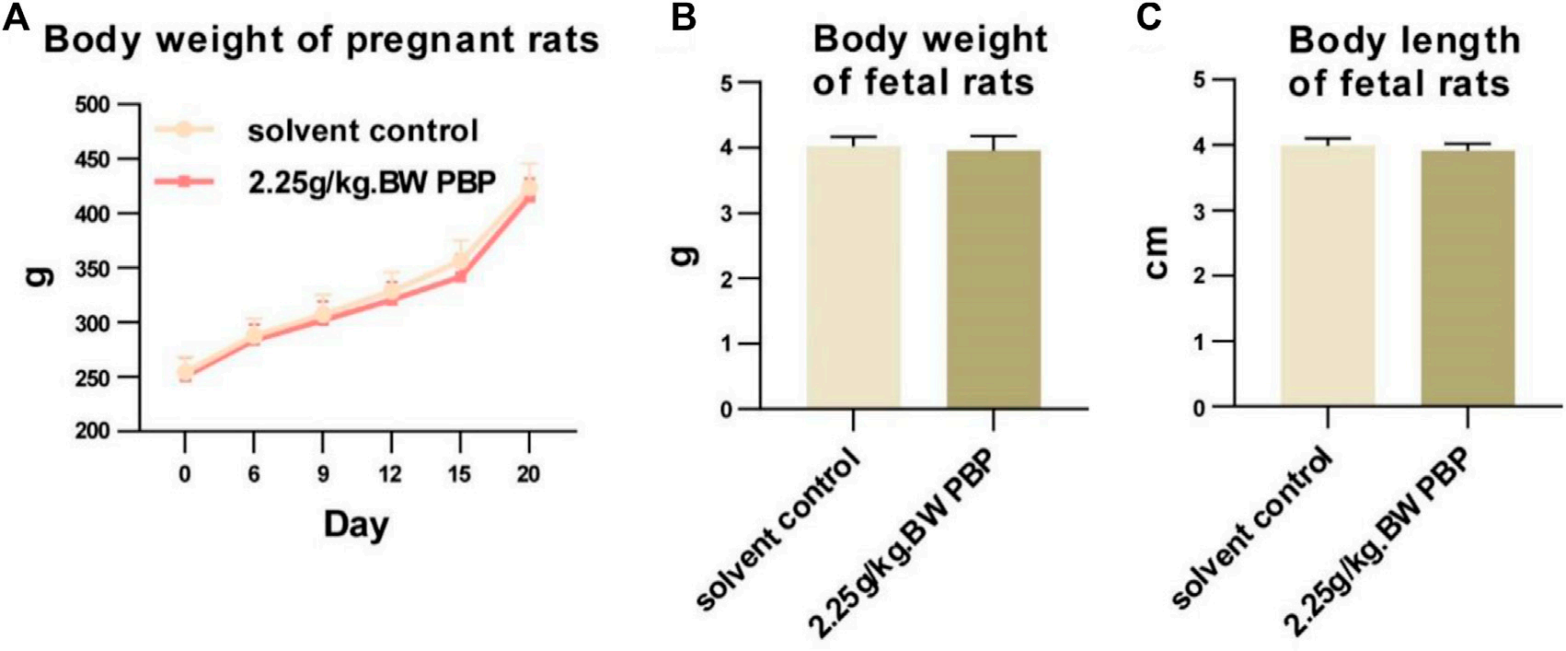
Teratogenicity experiment. **(A)** Body weight of pregnant rats (days 0, 6, 9, 12, 15, and 20 of gestation); **(B** and **C)** Body weights and body lengths of fetal rats.

A teratogenicity test was conducted to evaluate whether PBP has a toxic effect on fertilization and embryonic development. During this test, no signs of poisoning were observed, and the weight gain, and numbers of corpora lutea, premature stillbirths, late stillbirths, live births, and embryo implantation counts of pregnant rats in each dose group were not significantly different from those in the solvent control group (*p* > 0.05, Supplementary Tables S10–S12). The weight, body length, appearance, skeletal development, and internal organs of fetal rats in the dose groups were also not significant different from those of the fetal rats in the solvent control group (*p* > 0.05, Figure 3; Supplementary Tables S13–S15). Therefore, within the range of 0.75–2.25 g/kg BW, PBP had no toxic teratogenic effects.

## 4 Discussion

As a traditional Chinese medicine, PBP has been widely used for treating a range of diseases such as cough, asthma, constipation, dysentery, and boils. In addition to its applications in gastrointestinal, respiratory, and skin diseases, the potential of PBP in diseases involving lipid and glucose metabolism also warrants attention (He et al., 2011; He et al., 2017; Huang et al., 2019; Zheng et al., 2021a; Guan et al., 2022). Although the anti-inflammatory and hypoglycemic effects of PBP have been reported for many years, the components and side effects of PBP have not been well elucidated. There is thus a need to perform analyses of the components and toxicity of PBP.

According to the Chinese Pharmacopoeia, the minimum requirement for total THDCA content in medicinal-grade PBP is 2%. In the PBP used in our study, this content level was 5.1%. As mentioned above, HCA species are the most abundant substances in PBP, followed by CDCA species. In a previous study, it was asserted that HCA species can lower blood sugar levels (Chavez-Talavera et al., 2020; Zheng et al., 2021a). CDCA species significantly reduce cholesterol absorption *in vivo* and reduce the intestinal uptake of cholesterol by inhibiting the expression of cholesterol transport proteins. Additionally, it was reported that CDCA further reduces cholesterol absorption by regulating the composition and function of the intestinal microbial community (Ponz de Leon et al., 1979). Therefore, the regulatory role of PBP in human metabolism and its potential in other diseases warrant further investigation.

In the toxicity evaluation, we first performed a 14-day acute toxicity test to assess the safety of PBP. The aim here was to determine the acute toxic dose and the need for further toxicological studies. At the dose of 15 g/kg BW, no clinical abnormality or mortality was observed in the mice. Thus, the 14-day acute oral toxicity test showed that the LD_50_ of PBP in mice was >15 g/kg BW. According to the acute toxic dose classification of the National Food Safety Standard (GB 15193), a substance subjected to an acute oral test is considered practically nontoxic when its dose reaches 5 g/kg BW; therefore, PBP is nontoxic to the human body.

A subchronic oral toxicity test was performed using the maximum dose, which was 100 times higher than the expected human intake. The recommended human intake of PBP used in this test was converted to 0.0075 g/kg BW day. Thus, a dose of 0.75 g/kg BW day was selected for this test. A 90-day subchronic toxicity test was conducted to assess subchronic toxicity upon long-term exposure of PBP. In the test, no abnormal behavior, changes in diet and water intake, and weight, or pathological changes in organs were observed in the range of PBP of 0.75–2.25 g/kg BW day.

The same dose was also applied for the teratogenicity test. The teratogenicity test was conducted to assess the effects of drugs on fetal development. No impairment of maternal fertility or abnormal fetal development was observed in the rats. The results showed that there was no developmental toxicity in the PBP dose range of 0.75–2.25 g/kg BW.

The guidelines of the International Conference on Harmonization S2 (R1) recommend the Ames test, micronucleus test, and spermatogonial chromosomal aberration test to predict drug toxicity (Galloway, 2017). The Ames test can predict 70%–90% of carcinogenic substances, and assess the ability of drugs to cause mutation (Zeiger, 2019). Meanwhile, the micronucleus test and spermatogonial chromosomal aberration test were conducted to evaluate the ability of PBP to induce chromosomal damage and germ cell toxicity. According to the National Food Safety Standard (GB 15193), the highest concentration used for the Ames test should be 5,000 μg/plate. In the present study, a maximum dose of 10 g/kg BW was employed for the micronucleus test and spermatogonial chromosome mutation test. The negative results showed that PBP does not have mutagenic potential again genes, chromosomes, and reproductive cells at experimental doses.

## 5 Conclusion

Based on the National Food Safety Standard (GB 15193), it was determined that up to a dose of 15 g/kg BW, within the dose range of 50–5,000 μg/plate, and within the dose range of 0–2.25 g/kg BW of PBP, there were no signs of acute or subchronic oral toxicity, teratogenicity, or toxicity related to genes, chromosomes, or reproductive cells. Therefore, at the administered experimental dose, PBP can be considered non-toxic.

## Data Availability

The original contributions presented in the study are included in the article/Supplementary Material, further inquiries can be directed to the corresponding author.

## Abbreviations

BALF, bronchoalveolar lavage fluid; BW, body weight; CDCA, chenodeoxycholic acid; CP, cyclophosphamide; GCDCA, glycochenodeoxycholic acid; GHCA, glycohyocholic acid; GHDCA, glycohyodeoxycholic acid; GLP-1, glucagon-like peptide 1; HCA, hyocholic acid; HDCA, hyodeoxycholic acid; IFN, interferon; IL, interleukin; PBP, porcine bile powder; PCEs, polychromatic erythrocytes; RBCs, red blood cells; SPF, specific-pathogen-free; TCDCA, taurochenodeoxycholic acid; TGR5, G protein-coupled receptor 5; Th, T-helper; THCA, taurochenodeoxycholic acid; THDCA, taurohyodeoxycholic acid
