# Supplementary material for: Toxicological evaluation of porcine bile powder in Kunming mice and Sprague–Dawley rats
Source: Front Pharmacol. 2024 Jul 8;15:1424940. doi: 10.3389/fphar.2024.1424940 (PMC11260644; doi:10.3389/fphar.2024.1424940)
Supplement: Supplementary file 1 [file Table1.docx]

Supplementary Material

Table S1:

The effect of porcine bile powder (PBP) on the weight of rats in 1-13 weeks (mean ± SD).

| Sex | Female | | | | Male | | | |
| --- | --- | --- | --- | --- | --- | --- | --- | --- |
| Dose(g/kg.BW) | Solvent control | 0.75 | 1.5 | 2.25 | Solvent control | 0.75 | 1.5 | 2.25 |
| Initial BW(g) | 71.70±4.80 | 71.30±4.70 | 70.80±4.70 | 70.50±5.90 | 70.10±6.00 | 70 .40±5.80 | 70 .40±5.70 | 69 .40±6.30 |
| Week 1(g) | 112.80±4.30 | 114.50±4.10 | 113.80±5.70 | 113.80±6.60 | 118.80±6 .90 | 120.70±6 .20 | 119.20±6 .30 | 117.90±5 .80 |
| Week 2(g) | 156.40±8.00 | 157.40±8.20 | 155.90±7.40 | 155.20±8.50 | 182.20±9.20 | 183.00±10.60 | 183.20±9.40 | 182.60±9 .30 |
| Week 3(g) | 184.10±10.60 | 185.3±12.30 | 181.90±11.70 | 184.80±10.30 | 247.60±11 .30 | 246.40±15.50 | 248.80±11 .90 | 246.70±14 .00 |
| Week 4(g) | 211.50±13.10 | 210.70±10.00 | 205.60±13.50 | 212.70±11.50 | 309.00±15.90 | 315.30±21.40 | 304.90±21.60 | 307.20±18.20 |
| Week 5(g) | 234.60±14.60 | 233.30±13.40 | 229.10±14.90 | 235.00±14.00 | 367.90±18 .50 | 372.30±21 .8 | 360.40±23 .50 | 365.30±22 .50 |
| Week 6(g) | 257.20±14.50 | 255.60±17.20 | 249.90±15.70 | 257.30±17.90 | 412.70±20 .50 | 419.40±21 .30 | 406.80±24 .50 | 412.10±27 .80 |
| Week 7(g) | 276.60±16.10 | 274.40±15.80 | 267.50±15.20 | 277.00±14.40 | 453.60±27 .90 | 456.10±25 .70 | 443.30±33 .30 | 449.60±35 .90 |
| Week 8(g) | 289.50±16.60 | 291.60±16.10 | 281.90±15.30 | 290.40±14.90 | 484.60±31 .80 | 487.60±23 .40 | 473.08±34 .40 | 480.40±35 .20 |
| Week 9(g) | 298.90±16.70 | 305.30±16.20 | 292.40±16.00 | 299.20±16.70 | 503.90±31 .20 | 511.50±27 .20 | 495.60±38 .60 | 502.90±37 .80 |
| Week 10(g) | 307.00±17.30 | 313.40±18.10 | 300.20±17.40 | 307.20±17.90 | 524.90±37 .40 | 529.40±31 .20 | 517.90±39 .80 | 524.70±44 .40 |
| Week 11(g) | 313.70±17.50 | 320.90±18.60 | 307.60±17.40 | 314.30±20.10 | 546.40±35 .00 | 546.40±32 .70 | 539.80±43 .40 | 545.30±40 .80 |
| Week 12(g) | 320.10±16.60 | 327.00±16.10 | 314.10±16.60 | 320.40±18.50 | 565.30±38.50 | 562.90±37.50 | 557.50±39.90 | 565.00±39.80 |
| Week 13(g) | 326.10±17.20 | 331.70±19.10 | 319.90±16.10 | 326.40±18.60 | 582.30±30.10 | 577.90±35 .30 | 574.00±42 .30 | 585.00±36 .60 |
| Total BW gain (g) | 254.40±12.68 | 260.40±14.59 | 249.10±12.93 | 255.90±12.83 | 521.83±21.62 | 505.67±29.93 | 493.95±29.9 | 513.71±37.2 |
| Fasting BW(g) | 309.03±17.50 | 315.00±18.20 | 303.10±17.30 | 310.20±19.60 | 562.10±34.90 | 568.4±33.40 | 556.50±44.20 | 561.80±35.40 |

Table S2:

The effect of PBP on the food consumption of rats in 1-13 weeks (mean ± SD).

| Sex | Female | | | | Male | | | |
| --- | --- | --- | --- | --- | --- | --- | --- | --- |
| Dose (g/kg.BW) | Solvent control | 0.75 | 1.5 | 2.25 | Solvent control | 0.75 | 1.5 | 2.25 |
| Week 1 (g) | 92.9±5.0 | 93.0±5.8 | 93.2±4.5 | 92.4±6.2 | 99.9±7.1 | 100.8±8.2 | 97.1±8.0 | 99.6±8.2 |
| Week 2 (g) | 118.1±10.0 | 117.1±8.1 | 119.4±9.0 | 118.5±8.4 | 147.2±8.1 | 147.2±9.2 | 150.9±9.9 | 147.9±8.3 |
| Week 3 (g) | 128.0±10.8 | 129.3±11.7 | 130.5±10.4 | 128.3±10.3 | 173.4±11.3 | 175.2±11.0 | 173.2±10.9 | 170.5±10.2 |
| Week 4 (g) | 146.1±11.7 | 144.4±11.7 | 151.7±12.0 | 145.4±11.3 | 200.1±11.6 | 207.5±10.6 | 193.5±11.5 | 201.4±10.5 |
| Week 5 (g) | 157.9±9.8 | 160.0±10.5 | 154.3±13.7 | 156.7±11.2 | 209.9±11.4 | 201.6±12.8 | 204.0±12.6 | 210.7±12.1 |
| Week 6 (g) | 163.7±10.6 | 164.5±9.2 | 159.7±10.1 | 162.6±11.0 | 220.1±11.1 | 216.3±12.0 | 221.3±11.1 | 224.7±11.7 |
| Week 7 (g) | 168.6±10.1 | 166.7±12.6 | 167.7±12.2 | 165.8±10.6 | 249.4±10.3 | 240.3±10.2 | 244.6±9.5 | 247.4±9.9 |
| Week 8 (g) | 172.3±11.4 | 177.5±10.2 | 171.4±11.8 | 173.6±10.9 | 270.8±9.3 | 276.7±10.9 | 264.8±11.7 | 269.4±10.6 |
| Week 9 (g) | 177.6±8.9 | 181.4±9.8 | 180.8±10.3 | 177.3±8.6 | 278.3±9.4 | 277.0±9.7 | 279.5±11.1 | 277.7±10.8 |
| Week 10 (g) | 180.4±10.5 | 187.1±9.9 | 176.4±9.3 | 181.0±10.1 | 297.1±10.6 | 291.4±10.8 | 297.4±9.5 | 292.0±9.7 |
| Week 11 (g) | 186.0±10.8 | 190.9±10.4 | 185.3±11.3 | 186.2±10.8 | 309.2±10.0 | 309.8±11.0 | 303.2±10.2 | 300.0±9.5 |
| Week 12 (g) | 192.9±10.9 | 198.3±11.6 | 188.0±11.3 | 193.4±12.6 | 312.6±10.6 | 322.9±10.1 | 319.9±11.7 | 313.8±8.0 |
| Week 13 (g) | 196.9±12.6 | 203.2±13.2 | 200.9±12.3 | 197.1±13.1 | 318.9±10.7 | 327.6±10.5 | 324.3±12.9 | 318.1±10.1 |

Table S3:

The effect of PBP on the food utilization of rats in 1-13 weeks (mean ± SD).

| Sex | Female | | | | Male | | | |
| --- | --- | --- | --- | --- | --- | --- | --- | --- |
| Dose (g/kg.BW) | Solvent control | 0.75 | 1.5 | 2.25 | Solvent control | 0.75 | 1.5 | 2.25 |
| Week 1(%) | 44.3±1.7 | 46.7±3.8 | 46.2±3.5 | 47.1±3.9 | 49.3±4.1 | 50.8±6.0 | 52.8±4.3 | 61.8±6.1 |
| Week 2(%) | 37.2±4.6 | 37.0±6.2 | 35.4±3.0 | 35.0±2.3 | 43.2±3.0 | 42.6±5.9 | 42.6±3.9 | 43.9±3.8 |
| Week 3(%) | 21.6±2.8 | 21.8±4.5 | 20.3±7.1 | 23.2±2.8 | 37.9±3.1 | 36.4±4.6 | 38.0±3.3 | 37.8±5.5 |
| Week 4(%) | 18.9±3.3 | 17.7±3.3 | 15.8±6.1 | 19.3±2.6 | 30.8±4.4 | 33.2±5.7 | 29.0±5.9 | 30.1±3.3 |
| Week 5(%) | 14.8±4.0 | 14.3±3.8 | 15.2±3.3 | 14.2±1.8 | 28.1±4.8 | 28.4±5.5 | 27.5±5.4 | 27.7±7.6 |
| Week 6(%) | 13.9±2.1 | 13.5±3.6 | 13.2±3.7 | 13.8±3.3 | 20.5±3.6 | 21.8±2.6 | 21.0±3.1 | 21.0±5.9 |
| Week 7(%) | 11.5±1.8 | 11.3±1.9 | 10.6±3.2 | 11.9±3.1 | 16.4±3.9 | 15.4±4.3 | 14.8±5.9 | 15.1±4.5 |
| Week 8(%) | 7.5±2.6 | 9.8±3.5 | 8.3±3.2 | 7.7±2.4 | 11.5±3.6 | 11.4±2.0 | 11.5±3.1 | 11.5±4.7 |
| Week 9(%) | 5.3±2.3 | 7.5±3.2 | 5.8±3.0 | 4.9±4.7 | 7.0±4.8 | 8.6±2.3 | 7.8±7.0 | 8.1±3.9 |
| Week 10(%) | 4.5±3.4 | 4.3±2.7 | 4.5±4.5 | 4.6±6.0 | 7.1±5.1 | 6.1±4.3 | 7.5±4.9 | 7.5±4.5 |
| Week 11(%) | 3.7±2.9 | 4.0±5.0 | 4.1±3.8 | 3.9±3.1 | 6.9±4.4 | 5.5±6.6 | 7.2±6.0 | 6.9±5.3 |
| Week 12(%) | 3.3±4.9 | 3.2±5.3 | 3.5±2.7 | 3.4±5.1 | 6.0±2.8 | 5.1±4.2 | 5.6±5.7 | 6.4±5.3 |
| Week 13(%) | 2.9±4.1 | 2.3±2.4 | 2.8±4.0 | 3.2±5.2 | 5.3±4.6 | 4.6±2.3 | 5.1±4.6 | 6.2±7.5 |
| Total food utilization (%) | 12.2±0.6 | 12.3±0.7 | 12.0±0.7 | 12.3±0.8 | 16.6±0.8 | 16.4±1.0 | 16.4±1.3 | 16.8±1.0 |

Table S4:

Organ weight and the relative organ weight of rats (mean ± SD).

|  | Parameters | Organ | Dose(g/kg.BW) | |  |  |
| --- | --- | --- | --- | --- | --- | --- |
|  |  |  | Solvent control | 0.75 | 1.5 | 2.25 |
| Female | Organ weight（g） | Brain | 1.96±0.06 | 1.93±0.09 | 1.94±0.09 | 1.91±0.05 |
|  |  | Heart | 0.97±0.09 | 1.05±0.10 | 1.05±0.14 | 0.97±0.13 |
|  |  | Thymus | 0.38±0.10 | 0.41±0.08 | 0.37±0.06 | 0.38±0.06 |
|  |  | Adrenal gland | 0.07±0.01 | 0.07±0.01 | 0.06±0.01 | 0.06±0.01 |
|  |  | Liver | 8.59±1.12 | 8.55±1.23 | 8.07±1.07 | 8.08±1.18 |
|  |  | Kidney | 1.96±0.17 | 1.86±0.21 | 1.94±0.22 | 1.86±0.17 |
|  |  | Spleen | 0.58±0.12 | 0.55±0.11 | 0.58±0.07 | 0.60±0.12 |
|  |  | Uterus | 0.82±0.16 | 0.70±0.13 | 0.70±0.12 | 0.91±0.46 |
|  |  | Ovary | 0.14±0.04 | 0.14±0.04 | 0.15±0.04 | 0.14±0.05 |
|  | Relative organ weight（%） | Brain | 0.60±0.04 | 0.58±0.04 | 0.61±0.05 | 0.59±0.03 |
|  |  | Heart | 0.30±0.02 | 0.32±0.04 | 0.33±0.06 | 0.30±0.04 |
|  |  | Thymus | 0.12±0.03 | 0.12±0.02 | 0.12±0.02 | 0.12±0.02 |
|  |  | Adrenal gland | 0.02±0.00 | 0.02±0.00 | 0.02±0.00 | 0.02±0.00 |
|  |  | Liver | 2.63±0.28 | 2.58±0.35 | 2.52±0.27 | 2.49±0.43 |
|  |  | Kidney | 0.60±0.05 | 0.56±0.08 | 0.61±0.08 | 0.57±0.07 |
|  |  | Spleen | 0.18±0.04 | 0.17±0.03 | 0.18±0.02 | 0.18±0.04 |
|  |  | Uterus | 0.25±0.05 | 0.21±0.04 | 0.22±0.04 | 0.28±0.15 |
|  |  | Ovary | 0.04±0.01 | 0.04±0.01 | 0.05±0.01 | 0.04±0.02 |
| Male | Organ weight（g） | Brain | 2.10±0.06 | 2.04±0.08 | 2.00±0.09 | 2.06±0.11 |
|  |  | Heart | 2.10±0.06 | 2.04±0.08 | 2.00±0.09 | 2.06±0.11 |
|  |  | Thymus | 1.49±0.16 | 1.39±0.14 | 1.40±0.18 | 1.42±0.13 |
|  |  | Adrenal gland | 0.49±0.05 | 0.45±0.07 | 0.50±0.09 | 0.45±0.10 |
|  |  | Liver | 0.056±0.009 | 0.058±0.012 | 0.059±0.007 | 0.059±0.006 |
|  |  | Kidney | 11.88±1.19 | 11.88±1.18 | 12.21±1.46 | 12.92±1.42 |
|  |  | Spleen | 3.30±0.38 | 2.78±0.33 | 3.02±0.34 | 3.12±0.56 |
|  |  | Testicles | 0.89±0.14 | 0.87±0.13 | 0.90±0.11 | 0.97±0.18 |
|  |  | Epididymis | 3.55±0.56 | 3.76±0.26 | 3.58±0.26 | 3.44±0.35 |
|  | Relative organ weight （%) | Brain | 0.36±0.02 | 0.35±0.03 | 0.35±0.03 | 0.35±0.02 |
|  |  | Heart | 0.36±0.02 | 0.35±0.03 | 0.35±0.03 | 0.35±0.03 |
|  |  | Thymus | 0.26±0.03 | 0.24±0.02 | 0.25±0.04 | 0.24±0.02 |
|  |  | Adrenal gland | 0.08±0.01 | 0.08±0.01 | 0.09±0.01 | 0.08±0.02 |
|  |  | Liver | 0.01±0.00 | 0.01±0.00 | 0.01±0.00 | 0.01±0.00 |
|  |  | Kidney | 2.05±0.25 | 2.06±0.21 | 2.14±0.27 | 2.22±0.30 |
|  |  | Spleen | 0.57±0.08 | 0.48±0.06 | 0.53±0.08 | 0.53±0.08 |
|  |  | Testicles | 0.15±0.03 | 0.15±0.03 | 0.16±0.02 | 0.17±0.03 |
|  |  | Epididymis | 0.61±0.10 | 0.65±0.05 | 0.63±0.07 | 0.59±0.07 |

Table S5:

The effect of PBP on blood biochemical index (mean ± SD).

Alanine aminotransferase (ALT), methyltransferase (AST), total protein (TP), albumin (ALB), albumin/globulin (ALB/Glo), creatinine (Crea), urea (Urea), total cholesterol (TC), triglyceride (TG), blood glucose (Glu), alkaline phosphatase (ALP), glutamyl transpeptidase (GGT), chloride (Cl), potassium (K), sodium (Na).

| Sex | Female | | | | Male | | | |
| --- | --- | --- | --- | --- | --- | --- | --- | --- |
| Dose(g/kg.BW) | Solvent control | 0.75 | 1.5 | 2.25 | Solvent control | 0.75 | 1.5 | 2.25 |
| ALT(U/L) | 31.8±15.1 | 30.8±14.3 | 31.6±17.2 | 31.3±16.7 | 32.2±5.5 | 33.0±9.0 | 36.6±7.7 | 36.0±7.9 |
| AST(U/L) | 142.2±59.5 | 139.6±49.7 | 147.8±57.5 | 146.5±40.0 | 153.9±38.2 | 145.1±38.8 | 166.5±36.9 | 166.2±59.0 |
| TP(g/L) | 70.7±3.0 | 71.6±5.8 | 71.1±6.1 | 70.0±5.0 | 61.6±2.8 | 63.5±2.7 | 62.3±2.6 | 63.2±2.2 |
| ALB(g/L) | 38.6±1.5 | 39.5±3.1 | 38.9±3.6 | 37.8±2.9 | 32.9±1.0 | 32.3±1.0 | 32.5±1.6 | 32.2±1.0 |
| ALB/Glo | 0.35±0.01 | 0.36±0.01 | 0.35±0.01 | 0.35±0.01 | 0.35±0.01 | 0.34±0.01 | 0.34±0.01 | 0.35±0.05 |
| Crea (μmol/L) | 41.2±5.4 | 45.3±7.8 | 46.0±6.5 | 45.8±5.4 | 37.6±6.2 | 38.6±4.6 | 38.7±4.7 | 40.4±6.2 |
| Urea(mmol/L) | 6.12±0.92 | 6.62±0.99 | 7.04±1.25 | 6.88±1.14 | 6.33±1.49 | 6.72±1.47 | 6.48±1.55 | 6.17±1.37 |
| TC (mmol/L) | 2.50±0.55 | 2.21±0.50 | 2.41±0.58 | 2.32±0.31 | 1.89±0.20 | 1.86±0.29 | 1.94±0.29 | 1.83±0.40 |
| TG (mmol/L) | 0.49±0.19 | 0.72±0.37 | 0.50±0.13 | 0.49±0.10 | 0.46±0.21 | 0.54±0.20 | 0.49±0.17 | 0.60±0.26 |
| Glu(mmol/L) | 6.71±0.60 | 6.79±0.58 | 6.92±0.58 | 6.83±0.52 | 6.94±0.58 | 6.84±0.49 | 6.78±0.64 | 6.84±0.73 |
| ALP(U/L) | 49.6±12.9 | 42.7±12.3 | 49.3±22.3 | 56.5±10.4 | 157.5±66.8 | 152.8±59.6 | 140.0±59.0 | 144.4±51.1 |
| GGT(U/L) | 0.61±0.41 | 0.57±0.40 | 0.45±0.40 | 0.53±0.66 | 0.73±0.85 | 0.68±0.67 | 0.71±0.72 | 0.67±0.64 |
| Cl(mmol/L) | 102.5±1.6 | 102.6±1.6 | 102.4±1.1 | 102.2±1.5 | 102.9±1.1 | 102.8±1.8 | 101.9±0.8 | 102.2±1.0 |
| K(mmol/L) | 6.51±0.98 | 6.73±0.84 | 7.04±0.96 | 6.51±0.94 | 7.00±0.74 | 7.19±0.85 | 7.33±0.65 | 6.95±0.69 |
| Na(mmol/L) | 149.4±1.3 | 148.9±1.8 | 148.6±1.7 | 149.1±1.4 | 150.3±2.5 | 151.1±1.8 | 149.9±2.7 | 148.9±1.8 |

Table S6:

Effect of PBP on hematological indicators (mean ± SD).

White blood cell (WBC), red blood cell (RBC), hemoglobin (HGB), platelet (PLT), red blood cell pressure (HCT), lymphocyte (Lymph), neutrophil (Neut), monocyte (Mono), eosinophil (Eos), basophil (Baso), prothrombin time (PT), activated partial thromboplastin time (APTT).

| Sex | Female | | | | Male | | | |
| --- | --- | --- | --- | --- | --- | --- | --- | --- |
| Dose(g/kg.BW) | Solvent control | 0.75 | 1.5 | 2.25 | Solvent control | 0.75 | 1.5 | 2.25 |
| WBC(×109/L) | 3.35±1.26 | 4.13±0.61 | 3.66±0.95 | 4.73±1.97 | 6.33±1.76 | 7.17±1.24 | 8.63±2.54 | 7.64±2.27 |
| RBC(×1012/L) | 7.04±0.33 | 7.01±0.64 | 7.35±0.33 | 7.30±0.50 | 8.00±0.32 | 8.15±0.32 | 8.44±0.41 | 8.17±0.31 |
| HGB(g/L) | 142.9±5.5 | 142.5±6.8 | 146.1±7.1 | 145.9±9.2 | 157.8±6.4 | 161.0±7.7 | 162.6±8.0 | 158.4±5.5 |
| PLT(×109/L) | 1014.8±140.4 | 1005.6±133.6 | 1055.3±132.0 | 1058.5±132.1 | 967.4±108.2 | 974.9±108.6 | 972.7±100.7 | 1052.7±120.5 |
| HCT (%) | 35.4±1.4 | 34.6±2.8 | 36.4±1.7 | 35.9±1.9 | 38.1±1.6 | 39.0±1.9 | 39.3±1.7 | 38.3±1.7 |
| Lymph (%) | 71.69±5.55 | 67.43±7.58 | 71.99±8.69 | 75.50±6.44 | 63.24±10.1 | 67.56±8.14 | 66.48±6.97 | 64.35±7.17 |
| Neut (%) | 25.12±5.33 | 29.26±7.55 | 24.73±7.89 | 20.99±5.89 | 31.44±10.27 | 27.42±8.07 | 28.85±6.94 | 30.78±6.12 |
| Mono (%) | 3.05±0.98 | 3.14±1.37 | 3.14±1.67 | 3.23±1.03 | 5.14±0.83 | 4.84±1.78 | 4.44±0.71 | 4.61±1.45 |
| Eos (%) | 0.09±0.12 | 0.16±0.28 | 0.13±0.16 | 0.27±0.24 | 0.18±0.13 | 0.18±0.15 | 0.23±0.13 | 0.26±0.20 |
| Baso (%) | 0.05±0.10 | 0.01±0.03 | 0.01±0.03 | 0.01±0.03 | 0 | 0 | 0 | 0 |
| PT (s) | 15.7±0.9 | 15.5±0.6 | 16.0±0.7 | 16.2±0.6 | 14.2±5.0 | 17.8±6.4 | 18.2±6.1 | 17.2±3.2 |
| APTT (s) | 23.6±4.3 | 23.1±3.7 | 21.9±4.0 | 25.9±4.9 | 24.7±4.9 | 24.2±4.8 | 24.7±4.7 | 24.0±3.6 |

Table S7:

Effect of PBP on urinary index (mean ± SD).

| Sex | Dose (g/kg.BW) | Number of animals examined | Urine protein  (g/L) | Density | pH | Glucose (mmol/L) | Occult blood test (Cell/uL) |
| --- | --- | --- | --- | --- | --- | --- | --- |
| Female | 2.25 | 10 | 0 | 1.015±0.003 | 6.05±0.44 | 0 | 0 |
|  | 1.50 | 10 | 0 | 1.016±0.004 | 6.05±0.50 | 0 | 0 |
|  | 0.75 | 10 | 0 | 1.013±0.003 | 6.00±0.33 | 0 | 0 |
|  | Solvent control | 10 | 0 | 1.013±0.003 | 6.05±0.44 | 0 | 0 |
| Male | 2.25 | 10 | 0 | 1.013±0.004 | 6.10±0.21 | 0 | 0 |
|  | 1.50  0.75 | 10 | 0 | 1.013±0.003 | 6.15±0.24 | 0 | 0 |
|  |  | 10 | 0 | 1.012±0.003 | 6.20±0.26 | 0 | 0 |
|  | Solvent control | 10 | 0 | 1.012±0.003 | 6.20±0.26 | 0 | 0 |

| Table S8:  Red blood cell micronucleus test.  Polychromatic erythrocyte (PCE), red blood cell (RBC), normochromic erythrocyte (NCE). Compared with the solvent control group, ** indicates P<0.01.     \| Sex \| Dose \| Number of animals examined \| Number of PCEs examined \| Number of micronuclear cells \| Micronucleus rate (‰) \| PCE \| NCE \| PCE/RBC \| \| --- \| --- \| --- \| --- \| --- \| --- \| --- \| --- \| --- \| \| (g/kg.BW) \|  \|  \|  \| $\bar{x}$±SD \| $\bar{x}$±SD \| $\bar{x}$±SD \| $\bar{x}$±SD \| \| Female \| 10.0 \| 5 \| 10000 \| 8 \| 0.80±0.27 \| 101.2±1.6 \| 98.8±1.6 \| 0.51±0.01 \| \| 5.0 \| 5 \| 10000 \| 11 \| 1.10±0.22 \| 100.6±2.4 \| 99.4±2.4 \| 0.50±0.01 \| \| 2.5 \| 5 \| 10000 \| 9 \| 0.90±0.22 \| 102.0±2.5 \| 98.0±2.5 \| 0.51±0.01 \| \| Solvent control \| 5 \| 10000 \| 10 \| 1.00±0.35 \| 100.8±2.2 \| 99.2±2.2 \| 0.50±0.01 \| \| Positive control \| 5 \| 10000 \| 194 \| 19.40±3.09** \| 99.4±3.2 \| 100.6±3.2 \| 0.50±0.02 \| \| Male \| 10.0 \| 5 \| 10000 \| 10 \| 1.00±0.35 \| 101.6±3.0 \| 98.4±3.0 \| 0.51±0.01 \| \| 5.0 \| 5 \| 10000 \| 11 \| 1.10±0.22 \| 102.0±2.5 \| 98.0±2.5 \| 0.51±0.01 \| \| 2.5 \| 5 \| 10000 \| 9 \| 0.90±0.42 \| 100.6±1.5 \| 99.4±1.5 \| 0.50±0.01 \| \| Solvent control \| 5 \| 10000 \| 10 \| 1.00±0.35 \| 101.0±2.0 \| 99.0±2.0 \| 0.51±0.01 \| \| Positive control \| 5 \| 10000 \| 213 \| 21.30±2.77** \| 99.8±1.8 \| 100.2±1.8 \| 0.50±0.01 \| |
| --- | --- | --- | --- | --- | --- | --- | --- | --- | --- | --- | --- | --- | --- | --- | --- | --- | --- | --- | --- | --- | --- | --- | --- | --- | --- | --- | --- | --- | --- | --- | --- | --- | --- | --- | --- | --- | --- | --- | --- | --- | --- | --- | --- | --- | --- | --- | --- | --- | --- | --- | --- | --- | --- | --- | --- | --- | --- | --- | --- | --- | --- | --- | --- | --- | --- | --- | --- | --- | --- | --- | --- | --- | --- | --- | --- | --- | --- | --- | --- | --- | --- | --- | --- | --- | --- | --- | --- | --- | --- | --- | --- | --- | --- | --- | --- | --- | --- | --- | --- |

Table S9:

Spermatogonia chromosomal aberration test.

Compared with the solvent control group, ** indicates P<0.01.

| Dose  (g/kg.BW) | Number of animals examined |  |  | Number of aberrant chromosomes in each type | | | | | |  |  | Number of aberrant chromosomes in total | Rate of chromosomal aberration  (%) |
| --- | --- | --- | --- | --- | --- | --- | --- | --- | --- | --- | --- | --- | --- |
|  |  | Break | Fragment | Multi-Kinetochore | cycle | | | Translon-cation | Non-Kinetochore | minute | others |  |  |
| 10.0 (24h) | 5 | 1 | 1 | 0 | 0 | 0 | | | 0 | 0 | 0 | 2 | 0.40±0.55 |
| 10.0 (48h) | 5 | 1 | 0 | 0 | 0 | | 0 | | 0 | 0 | 0 | 1 | 0.20±0.45 |
| 5.0 | 5 | 1 | 0 | 0 | 0 | | 0 | | 0 | 0 | 0 | 1 | 0.20±0.45 |
| 2.5 | 5 | 1 | 1 | 0 | 0 | | 0 | | 0 | 0 | 0 | 2 | 0.40±0.89 |
| Solvent control | 5 | 1 | 1 | 0 | 0 | | 0 | | 0 | 0 | 0 | 2 | 0.40±0.89 |
| Positive control | 5 | 25 | 8 | 0 | 0 | | 0 | | 0 | 5 | 0 | 38 | 7.60±0.89** |

Table S10:

Effect of PBP on pregnant rats (mean ± SD).

| Dose (g/kg.BW) | Number of pregnant rats examined | Days of pregnant | | | | | | Weight gain (g) | Net weight gain (g) |
| --- | --- | --- | --- | --- | --- | --- | --- | --- | --- |
|  |  | Day 0 | Day 6 | Day 9 | Day 12 | Day 15 | Day 20 |  |  |
| 2.25 | 18 | 250.5±17.1 | 283.6±14.3 | 301.7±17.5 | 320.6±16.4 | 341.7±20.8 | 415.0±16.9 | 131.4±13.3 | 48.9±17.6 |
| 1.50 | 17 | 249.2±17.4 | 282.4±16.5 | 296.5±18.0 | 318.3±20.0 | 347.7±20.9 | 409.1±23.9 | 126.7±22.3 | 49.5±15.9 |
| 0.75 | 17 | 247.4±14.2 | 280.6±16.0 | 299.9±19.2 | 317.7±21.5 | 347.1±20.2 | 417.5±22.8 | 136.9±11.6 | 57.1±17.0 |
| Solvent control | 17 | 254.4±14.2 | 288.0±15.8 | 307.4±17.7 | 328.4±17.5 | 356.2±19.0 | 423.7±22.0 | 135.7±15.8 | 49.5±14.3 |

Table S11:

Effect of PBP on pregnant rats (mean ± SD).

Compared with the solvent control, the P value > 0.05.

| Dose (g/kg.BW) | Number of rats examined | Number of pregnant rats | Percentage of pregnant rats (%) | Number of corpus luteum | Number of implantations | Number of live births | Weight of uterus-conjoined fetus (g) |
| --- | --- | --- | --- | --- | --- | --- | --- |
| 2.25 | 22 | 18 | 81.8 | 16.9±2.3 | 13.4±3.7 | 13.4±3.6(241) | 82.5±19.9 |
| 1.50 | 22 | 17 | 77.3 | 17.8±3.2 | 12.9±4.3 | 12.5±4.8(213) | 77.2±19.2 |
| 0.75 | 22 | 17 | 77.3 | 16.6±3.3 | 12.1±4.9 | 12.1±4.9(205) | 79.8±20.1 |
| Solvent control | 22 | 17 | 77.3 | 16.8±1.7 | 14.5±2.0 | 14.4±2.0(245) | 86.2±15.3 |

Table S12:

Effect of PBP on fetal mortality of pregnant rats (mean ± SD).

| Dose (g/kg.BW) | Number of pregnant rats examined | Number of the absorbed fetus | Number of early stillbirths | Number of late stillbirths | Number of pregnant rats with the absorbed fetus | Number of the absorbed fetus per litter (%) | Number of pregnant rats with early stillbirths | Number of early stillbirths per litter (%) | Number of pregnant rats with late stillbirths | Number of late stillbirths per litter (%) |
| --- | --- | --- | --- | --- | --- | --- | --- | --- | --- | --- |
| 2.25 | 18 | 1 | 0 | 0 | 1 | 5.56 | 0 | 0 | 0 | 0 |
| 1.50 | 17 | 2 | 0 | 0 | 1 | 5.88 | 0 | 0 | 0 | 0 |
| 0.75 | 17 | 1 | 0 | 0 | 1 | 5.88 | 0 | 0 | 0 | 0 |
| Solvent control | 17 | 2 | 0 | 0 | 2 | 11.76 | 0 | 0 | 0 | 0 |

Table S13:

Effect of PBP on the development of fetal rats (mean ± SD).

| Dose (g/kg.BW) | Number of pregnant rats examined | Average fetal rats weight (g) | The average body length of fetal rats (cm) | Number of deformities in appearance/number of litters | Percentage of deformities in appearance per litter (%) |
| --- | --- | --- | --- | --- | --- |
| 2.25 | 18 | 3.96±0.22 | 3.91±0.11 | 0 | 0 |
| 1.50 | 17 | 3.97±0.28 | 3.89±0.17 | 0 | 0 |
| 0.75 | 17 | 4.07±0.22 | 3.94±0.13 | 0 | 0 |
| Solvent control | 17 | 4.02±0.14 | 3.99±0.11 | 0 | 0 |

Table S14:

The effect of PBP on skeletal development of fetal rats (mean ± SD).

No abnormality was found in other items of bone examination.

| Dose (g/kg.BW)  Deformity category | 2.25 | 1.50 | 0.75 | Solvent control |
| --- | --- | --- | --- | --- |
| Number of live fetuses/number of litters | 125/18 | 112/17 | 106/17 | 127/17 |
| Number of sternum defects/number of litter | 13/6 | 14/5 | 13/5 | 15/6 |
| Rate of missing sternum/litter (%) | 33.3 | 29.4 | 29.4 | 35.3 |
| Number of xiphoid process defects/number of litters | 8/4 | 7/4 | 3/2 | 7/4 |
| Rate of missing xiphoid process /litter (%) | 22.2 | 23.5 | 11.8 | 23.5 |
| Number of hypoplasia of the ossification center of the occipital defects/number of litters | 6/3 | 4/2 | 5/2 | 3/3 |
| Rate of hypoplasia of the ossification center of the occipital/litter (%) | 16.7 | 11.8 | 11.8 | 17.6 |
| Number of rib abnormalities/number of litters | 0 | 0 | 0 | 0 |
| Rate of rib abnormalities/litter (%) | 0 | 0 | 0 | 0 |
| Number of dysplasia of the spine/number of litters | 0 | 0 | 0 | 0 |
| Rate of dysplasia of the spine/litter (%) | 0 | 0 | 0 | 0 |

Table S15:

Effect of PBP on fetal rat visceral development (mean ± SD)

| Dose (g/kg.BW) | Number of fetal rats examined | Number of visceral normal fetal rats/number of litters | Number of visceral abnormal fetal rats/number of litters | Percentage of visceral malformations per litter (%) |
| --- | --- | --- | --- | --- |
| 2.25 | 116 | 116/17 | 0 | 0 |
| 1.50 | 101 | 101/16 | 0 | 0 |
| 0.75 | 99 | 99/16 | 0 | 0 |
| Solvent control | 118 | 118/17 | 0 | 0 |


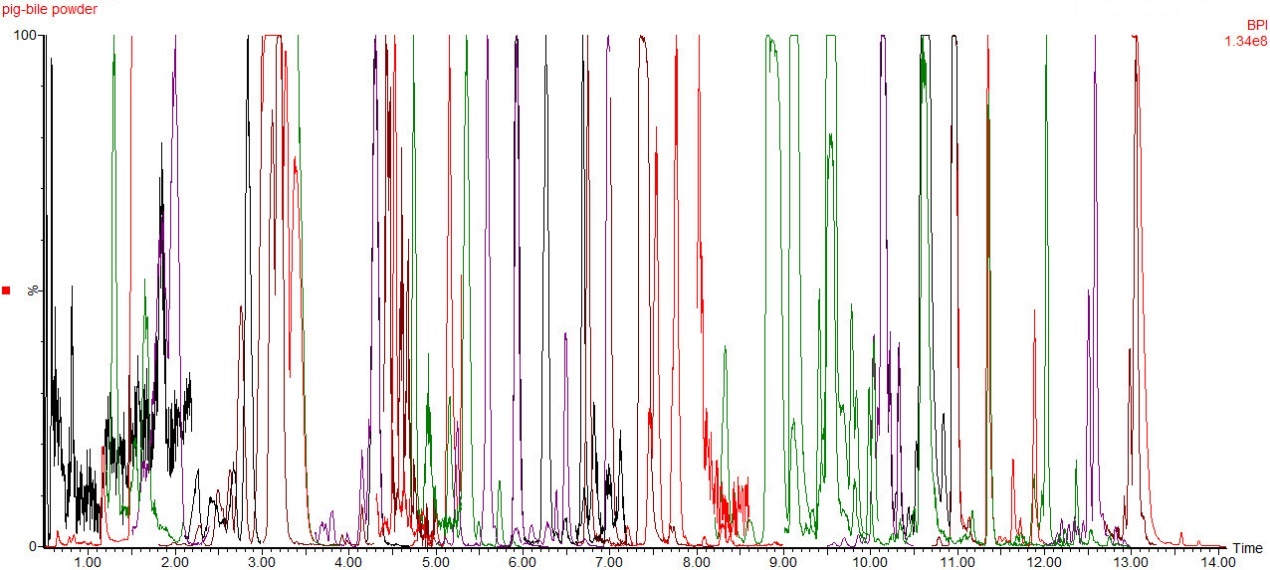


A

B


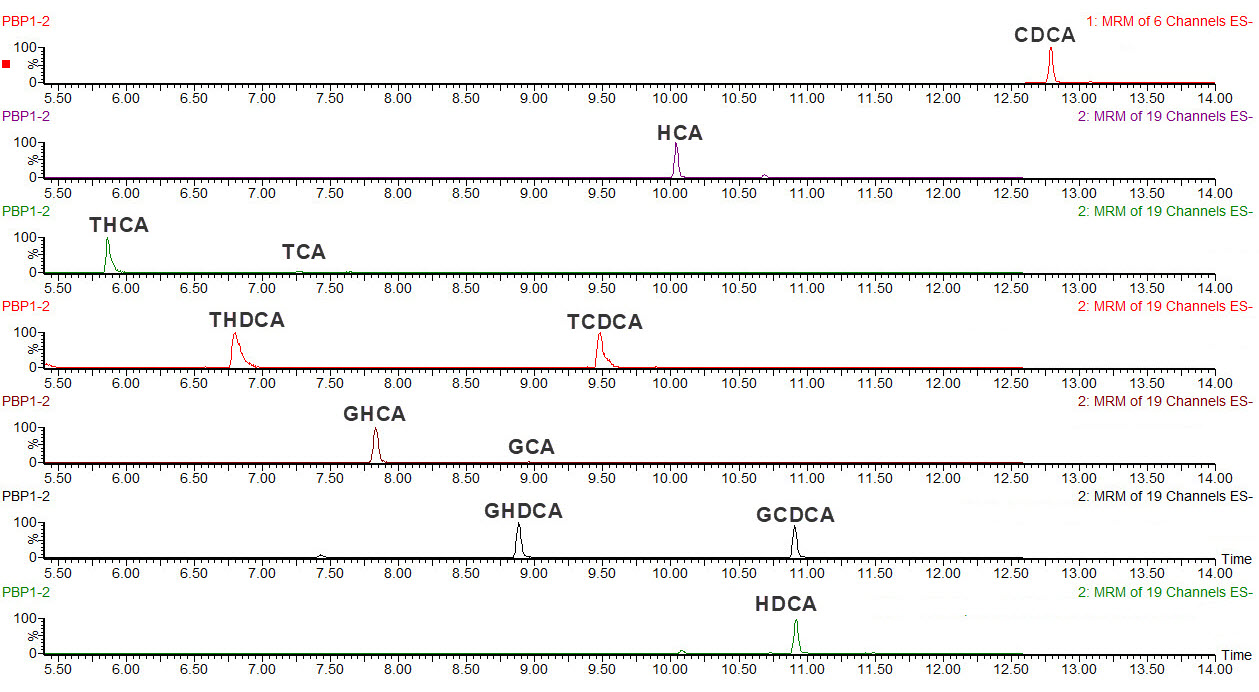


Figure S1. (A) Typical total ion chromatograms of all metabolites identified in pig bile powder. (B) Typical extracted ion chromatograms of bile acids in pig bile powder.
